# Microscopic Haematuria and Clinical Outcomes in Patients With Stage 3–5 Nondiabetic Chronic Kidney Disease

**DOI:** 10.1038/srep15242

**Published:** 2015-10-16

**Authors:** Hugo You-Hsien Lin, Chun-Yu Yen, Lee-Moay Lim, Daw-Yang Hwang, Jer-Chia Tsai, Shang-Jyh Hwang, Chi-Chih Hung, Hung-Chun Chen

**Affiliations:** 1 Division of Nephrology, Department of Internal Medicine, Kaohsiung Medical University Hospital, Kaohsiung Medical University; 2 Department of Internal Medicine, Kaohsiung Municipal Ta-Tung Hospital, Kaohsiung Medical University; 3 Graduate Institute of Medicine, College of Medicine, Kaohsiung Medical University, Kaohsiung, Taiwan; 4 Faculty of Renal Care, College of Medicine, Kaohsiung Medical University, Kaohsiung, Taiwan; 5 Institute of Population Sciences, National Health Research Institutes, Miaoli, Taiwan; 6 Department of Pediatrics, Taipei Veterans General Hospital, Taipei, Taiwan, R.O.C

## Abstract

Microscopic haematuria is proposed as a prognostic factor for renal outcomes in patients with glomerulonephritis. However, the role of haematuria in patients with advanced chronic kidney disease (CKD) or heavy proteinuria has not been investigated. We divided 1799 patients with stage 3–5 nondiabetic CKD into 3 groups according to the results from 3 urinalyses: no haematuria (0–2 red blood cells [RBCs]/hpf ≥2 times), mild haematuria (2–5 RBCs/hpf ≥2 times) and moderate haematuria (≥5–10 RBCs/hpf ≥2 times). The estimated glomerular filtration rate was 25.4 mL/min/1.73 m^2^, with a urine protein-to-creatinine ratio (UPCR) of 881 mg/g. The hazard ratios (HRs) of mild and moderate haematuria for end-stage renal disease (ESRD) were 1.28 (95% confidence interval [CI]: 1.05–1.56, P = 0.024) and 1.34 (95% CI: 1.03–1.74, P = 0.030), respectively. The HR of moderate haematuria for mortality was 1.56 (95% CI: 1.11–2.20, P = 0.011). According to subgroup analysis, the HR of moderate haematuria for ESRD in patients with a UPCR of <500 mg/g was more prominent than that in patients with a UPCR of ≥500 mg/g. Microscopic haematuria in patients with stage 3–5 nondiabetic CKD is associated with increased risks of ESRD and mortality.

Chronic kidney disease (CKD), an increasing global public health problem^1^, is associated with major critical sequelae, including mortality, end-stage renal disease (ESRD), and cardiovascular (CV) disease^2^. Currently, the most commonly used indicators of CKD progression are estimated glomerular filtration rate (eGFR) and proteinuria. Screening for proteinuria and haematuria by using a dipstick is a quick and effective method for detecting renal abnormalities. Microscopic haematuria (hereafter, “haematuria”) is frequently reported in patients with glomerular nephritis (GN) without proteinuria, such as thin basement membrane (TBM) disease, or with mild proteinuria, such as IgA nephropathy (IgAN). However, apart from its use in community screening for early GN, the prevalence and consequences of haematuria in patients with advanced-stage CKD or heavy proteinuria remain unknown.

Haematuria might be a risk factor for poor renal outcomes in patients with early-stage GN. In the general population, isolated haematuria without proteinuria has been associated with a high risk of ESRD after long-term follow-up; however, the incidence is as low as 0.3%^3^,^4^. Moreover, 15%–20% of patients with IgAN or other proliferative GN with isolated haematuria develop proteinuria^5^,^6^. Studies have considered haematuria as a risk factor for the progression of renal function and ESRD in patients with IgAN and other proliferative GN with mild proteinuria, even when pathological grading was also considered^7^,^8^,^9^,^10^,^11^. However, other studies have not confirmed or evaluated the relevance of haematuria in proliferative GN^12^,^13^,^14^. In patients with nephrotic syndrome, haematuria sometimes presents with nonproliferative GN^15^ and is considered to be associated with renal function progression and focal segmental glomerulosclerosis (FSGS)^16^. The prognostic value of haematuria for ESRD in nondiabetic patients with advanced-stage CKD or heavy proteinuria remains incompletely understood. In addition, haematuria can indicate glomerular basement membrane (GBM) injury and potentially harms renal tubules^17^,^18^. Therefore, in the present study, we hypothesised that haematuria is associated with ESRD and other clinical outcomes in patients with stage 3–5 nondiabetic CKD, assessing this hypothesis by examining an observational cohort of patients with CKD.

## Methods

### Participants and Measurements

From November 11, 2002, to May 31, 2009, a prospective observation study was conducted and 2 affiliated hospitals of Kaohsiung Medical University in Southern Taiwan; follow-up continued until May 31, 2010^19^. The integrated CKD care program for delaying dialysis included 3303 patients with stage 3–5 CKD. Of these patients, patients with a diabetes mellitus (DM) diagnosis based on the treatment administered or had a glycated hemoglobin level of ≥6.5% at the time of enrolment were excluded. Eventually, 1799 eligible participants were included in the study. The study protocol was approved by the Institutional Review Board of Kaohsiung Medical University Hospital, and all participants provided written informed consent to participate in this study. The methods were carried out in accordance with the approved guidelines.

The baseline variables of all participants included demographic data, comorbidities, medication history, lifestyle factors, physical examination findings, and laboratory data. Microscopic hematuria was defined as ≥2 to 5 and ≤25 to 50 red blood cells (RBCs) under high power field (RBCs/hpf) in three consecutive urinalysis after enrolment based on the laboratory grading system of our hospital: 0–2 (normal), 2–5, 5–10, 10–25, 25–50, and >50 RBCs/hpf. Samples exhibiting >25 white blood cells (WBCs)/hpf in urinalysis were excluded. Patient demographic data were recorded at the first visit, and medical history was recorded according to a chart review. Hypertension was defined on the basis of clinical diagnoses and medications prescribed. CV diseases were defined according to the clinical diagnoses of heart failure, acute or chronic ischemic heart disease, or cerebrovascular disease. Moreover, laboratory data were obtained at the first visit.

### Clinical Outcomes

Four clinical outcomes were accessed: ESRD, rapid renal function progression, all-cause mortality, and CV events. ESRD was defined as the initiation of haemodialysis, peritoneal dialysis, or renal transplantation. The initiation of ESRD was ascertained by a chart and catastrophic card review. Rapid renal function progression was defined when the eGFR slope was less than −5 mL/min/1.73 m^2^/year. The eGFR was defined by using the simplified Modification of Diet in Renal Disease Study equation: eGFR (mL/min/1.73 m^2^) = (186) × (serum creatinine ^−1.154^) × (age^−0.203^) × (C), where C is 0.742 for women, 1.212 for African American patients, and 1 for all other patients. The survival status and cause of death were determined by referencing death certificates, patient charts, and the National Death Index. CV events were ascertained by reviewing charts to identify hospitalisations for acute coronary syndrome, acute cerebrovascular disease, congestive heart failure, and peripheral arterial occlusion disease and death resulting from any of the aforementioned causes.

### Statistical Analysis

The statistical results of participant baseline characteristics were summarised and expressed as counts and percentages for the categorical data, means with standard deviation (SD), and medians with interquartile ranges (IQR) for the continuous variables with approximately normal distributions. Cox proportional hazards analysis was used to assess the relationship between haematuria and clinical outcomes. Moreover, multivariate logistic regression analysis was used to evaluate the relationship between haematuria and rapid renal function progression. The covariates were selected according to our previous studies, and the continuous variables with skewed distributions were log-transformed to obtain normal distributions^19^. The model was adjusted for age, sex, eGFR, log-transformed urine protein-to-creatinine ratio (UPCR), hypertension, CV disease, mean blood pressure, haemoglobin, albumin, body mass index (BMI), log-transformed cholesterol, log-transformed C-reactive protein (CRP), phosphorus, glomerulonephritis, tubulointerstitial nephritis, and hypertensive nephropathy. Furthermore, Cox survival analysis with prespecified subgroups was performed for all participants stratified according to age (65 years), sex, CVD, CKD stages, UPCR (500 and 1500 mg/g), hemoglobin (10 g/dL), albumin (3.5 g/dL), CRP (3 mg/L), and BMI (25 kg/m^2^). The interaction term was tested by adding a cross-product into the model. A P value < 0.05 was considered statistically significant. Statistical analysis was performed using the R 2·15·2 software (R Foundation for Statistical Computing, Vienna, Austria) and the Statistical Package for Social Sciences, Version 18.0, for Windows (SPSS Inc., Chicago, IL, USA).

## Results

### Characteristics and Clinical Outcomes According to Haematuria

The mean age of the 1799 patients was 62.6 ± 14.5 years, and 757 (42.1%) of the patients were women (Table 1). The eGFR was 25.4 ± 15.7 mL/min/1.73 m^2^, with a UPCR of 881 mg/g (IQR: 333–1766 mg/g). Overall, 1015 (56.4%) patients had hypertension, and 326 (18.1%) patients had CV disease. The patients were divided into 3 groups according to the severity of haematuria in 2 out of 3 consecutive urinalyses: Group 1, no haematuria (0-2 RBCs/hpf or ≥2–5 RBCs/hpf only once in 3 analyses); Group 2, mild haematuria (2–5 RBCs/hpf ≥2 times); and Group 3, moderate haematuria (≥5–10 RBCs/hpf ≥2 times). Other combinations of 2–5 RBCs/hpf once and ≥5–10 RBCs/hpf once were classified as mild haematuria.

The increase in Groups 1–3 was associated with a higher proportion of women; patients with stage 5 CKD; a stepwise increase in UPCR, white blood cell count, total cholesterol, phosphorus, and CRP; and a stepwise decrease in serum haemoglobin, albumin, and calcium (Table 1). After a median follow-up of 1157 days, the increment in Groups 1–3 was associated with rapid renal function progression, ESRD, mortality, and CV events.

### Factors Related to Mild or Moderate Haematuria

In multivariate logistic regression analysis, both the log-transformed UPCR and log-transformed CRP revealed higher odds ratios (ORs) for haematuria (Groups 2 and 3), whereas albumin revealed lower ORs for haematuria (Table 2). By contrast, eGFR and mean blood pressure (BP) were not associated with haematuria.

### Association of Haematuria with ESRD and Rapid Renal Function Progression

Overall, the disease condition of 507 patients (28.2%) progressed to ESRD. In the fully adjusted Cox proportional hazards model, a significant association was observed between haematuria and an increased risk of ESRD. The Group 2 and 3 patients exhibited a significantly higher risk of ESRD than did the Group 1 patients (hazard ratio [HR]: 1.28, 95% confidence interval [CI]: 1.05–1.56, P = 0.024 and 1.34, 95% CI: 1.03–1.74, P *=* 0.030, respectively; Table 3). The median renal function progression rate (eGFR slope) was −1.7 mL/min/1.73 m^2^/year. In the fully adjusted multivariate logistic regression model, a significant association was observed between haematuria and an increased risk of rapid renal function progression (eGFR slope less than −5 mL/min/1.73 m^2^/year). The ORs of Groups 2 and 3 were 1.45 (95% CI: 1.05–2.00, P = 0.023) and 1.54 (95% CI: 1.06–2.25, P = 0.023), respectively (Table 3).

### Association of Haematuria with All-Cause Mortality and Cardiovascular Events

Overall, 252 mortalities (14.0%) were recorded in our cohort during the follow-up period. In the fully adjusted Cox proportional hazards model, a significant association was observed between moderate haematuria and an increased risk of all-cause mortality (HR = 1.56, 95% CI: 1.11–2.20, P = 0.011) compared with no haematuria (Table 3).

In total, 361 CV events and mortalities (20.1%) were recorded during the follow-up period. In the fully adjusted Cox proportional hazards model, moderate haematuria was nonsignificantly associated with an increased risks of CV events and mortality (HR = 1.33, 95% CI: 0.97–1.82, P = 0.204; Table 3).

### Subgroup Analysis and Sensitivity Assessment: Association Between Haematuria and Clinical Outcomes

**Figure 1:**
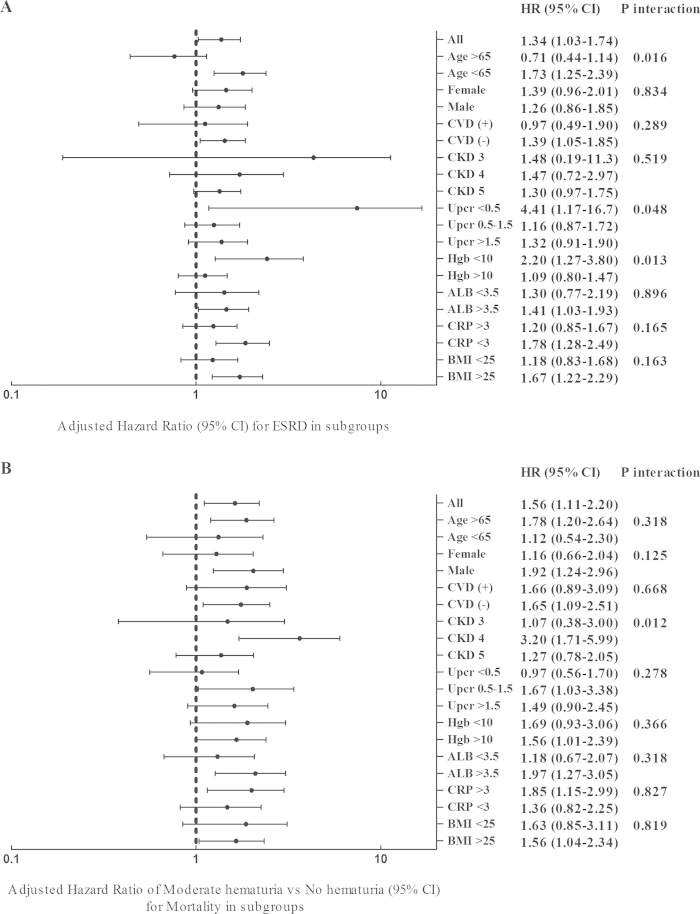
Forest tree plot of the HR per one-unit increase of RBCs for (1A) end-stage renal disease (ESRD) and (1B) rapid renal function progression.

Because haematuria was related to eGFR and UPCR, we further performed Cox regression analysis on the prespecified subgroups for ESRD and all-cause mortality (Table 4 and Fig. 1A). Age, UPCR, and haemoglobin, but not CKD stage, affected the association between haematuria and ESRD. The HR of moderate haematuria for ESRD in the subgroup with a UPCR <500 mg/g was 4.41 compared with 1.16 and 1.32 in the subgroups with UPCRs of 500–1500 mg/g and >1500 mg/g, respectively (Table 4 and Fig. 1A). In addition, CKD stage modified the association between haematuria and all-cause mortality. The HR of moderate haematuria for ESRD in the patient subgroup with stage 4 CKD was 3.20 compared with 1.07 and 1.27 in the patient subgroups with stage 3 and stage 5 CKD, respectively (Fig. 1B).

Moreover, the urine occult blood (UOB) measured using dipsticks was evaluated to confirm our results. The definition of haematuria according to UOB was UOB + to +++ for ≥2 times in 3 consecutive urinalyses. Because comparing the grading systems used for urine WBCs/hpf and UOB was challenging, these 2 measurements were dichotomously compared. In the fully adjusted Cox proportional hazards model, the risk of haematuria for ESRD revealed an HR of 1.29 (95% CI: 1.07–1.56, P = 0.007), and similarly, the risk of UOB for ESRD revealed an HR of 1.33 (95% CI: 1.01–1.75, P = 0.041) (Supplemental Table 1). Haematuria was associated with an increased risk of all-cause mortality (HR: 1.28, 95% CI: 0.98–1.66, P = 0.067), whereas no association was observed between UOB and all-cause mortality.

## Discussion

The present study investigated whether haematuria was associated with clinical outcomes in a stage 3–5 nondiabetic CKD cohort, revealing that haematuria was associated with proteinuria, hypoalbuminemia, and a high CRP. In addition, mild or moderate haematuria was significantly associated with increased risks of ESRD and rapid renal function progression, whereas moderate haematuria was associated with an increased risk of all-cause mortality. The association between haematuria and ESRD was more prominent in patients with a UPCR <500 mg/g.

Microscopic haematuria may be a clinical risk factor for renal outcomes in patients with biopsy-proven GN and mild proteinuria. Glomerular haematuria may indicate a defect in the GBM because of an abnormal GBM structure (thinning, irregularity, and disruption) or mesangial proliferation^17^,^20^. GBM diseases, such as TBM disease and Alport syndrome, and proliferative GN, such as IgAN, were the most common causes of isolated glomerular haematuria^21^. A previous study reported that isolated haematuria might result in the development of proteinuria in patients with IgAN and familial TBM^5^,^22^. Another study, conducted before angiotensin-converting enzyme (ACE) inhibitors were developed, involving a Dutch IgAN cohort, revealed that haematuria is a risk factor for ESRD^9^. This finding has been confirmed and explained in several other cohort studies performed after ACE inhibitors were developed^7^,^11^,^23^. A study in Japan involving a large IgAN cohort, the majority (86.7%) of patients in which had stage 1–3 CKD, reported that mild haematuria (1–29 RBCs/hpf) was associated with an increased risk of ESRD when all clinical and pathological factors were considered^10^. However, certain studies, including a cohort study in Japan, could not confirm the association of haematuria with ESRD^12^,^14^. However, haematuria is an underrecognised risk factor in most studies focusing on IgAN. According to our ongoing meta-analysis, approximately half of studies involving IgAN cohorts overlooked presenting haematuria in the descriptive results and analyses. In our renal biopsy registry, approximately one-third of the patients had IgAN (unpublished data). Thus, haematuria can be reasonably included as a potential risk factor in patients with nondiabetic CKD. Our study further suggests that haematuria should be considered in patients with stage 3–5 CKD, particularly in those with mild proteinuria.

Few studies have investigated the prognostic effects of haematuria in patients with other biopsy-proven proliferative GN or nephrotic syndromes. In patients with lupus nephritis, the incidence of haematuria was higher in the renal function impairment group than in the normal group^24^. In patients with pauciimmune GN, the development of microscopic haematuria during remission indicated glomerular injury^8^. However, the prognostic effects of haematuria regarding long-term renal outcomes have not been evaluated. Regarding nephrotic syndrome, haematuria is frequently observed in approximately 40% of patients with membranous nephropathy (MN)^25^ and FSGS, particularly in those with familial FSGS^15^, but is infrequent in patients with minimal change disease^26^. Reportedly, mesangial proliferation correlated with patients with nephrotic syndrome and haematuria^26^. The incidence of haematuria was lower in nephrotic syndrome cohorts, and only a few small-scale studies have discussed the prognostic effects of haematuria in nephrotic syndromes^13^,^16^,^25^. Regarding FSGS, haematuria is an independent predictor of stage 3 CKD^16^, whereas regarding MN, haematuria is not associated with developing ESRD^25^. In addition, in patients with nephrotic IgAN, haematuria is not a risk factor for developing ESRD^13^. However, the aforementioned studies were small-scale investigations. Consistent with the findings from such studies, the findings from our study suggest that haematuria might have less prognostic value in patients with advanced CKD and heavy proteinuria compared with its prognostic value in patients with mild proteinuria. This result can be explained by the strong correlation between haematuria and proteinuria, suggesting that the final pathologic pathway in nephrotic syndromes such as glomerulosclerosis might cause haematuria. Thus, the role of haematuria in patients with GN and heavy proteinuria warrants further exploration.

Little is known about the prevalence and consequences of haematuria in patients with other renal diseases of specific causes; studies on hypertension have indicated that in addition to GN, high blood pressure could be another mechanism of haematuria. Arteriolar nephrosclerosis is a type of characterised renal damage associated with malignant hypertension^27^. Haematuria has been reported in 30%–55% of patients with biopsy-proven hypertensive nephropathy^27^,^28^,^29^ and in 21% of patients with malignant hypertension^30^. Haematuria and proteinuria can occur in patients with high blood pressure and with or without other renal diseases^29^. The aforementioned literature suggests the haematuria presents only in patients whose blood pressure exceeds 180/100 mmHg, a threshold higher than that of proteinuria^27^. The prognostic effect of haematuria on dialysis was considered and demonstrated in only one study on malignant hypertension; both baseline creatinine and haematuria were associated with dialysis^30^. However, our data revealed no correlation between haematuria and mean blood pressure, likely because blood pressure was effectively controlled in this population. Whether a link exists between high blood pressure and haematuria in patients with advanced CKD and uncontrollable blood pressure warrants further examination.

Haematuria could be a risk factor for ESRD in the long-term follow-up of nonbiopsied patients. In a screening program including 107,192 people from Okinawa, Japan, haematuria was identified as a predictor of ESRD in the general population^3^. In a large nationwide cohort study, including 1.20 million people aged 16–25 years, conducted in Israel, persistent asymptomatic isolated haematuria (≥5 RBCs/hpf on 3 separate occasions) was strongly associated with ESRD^4^. Moreover, IgAN and hereditary nephritis were the most common causes amongst the patients who developed ESRD^4^. Conversely, another study involving a cohort comprising 177 570 people in California, the United States, and the dipstick haemoglobin test revealed nonsignificant trends of an increased risk of ESRD^31^. These data suggest that haematuria is a potential risk factor for ESRD in the general population in the absence of other pathological causes. Our study extends the findings that haematuria, measured using a microscope or dipstick, is a risk factor for ESRD development in patients with advanced nondiabetic CKD. The clinical application of these results is most useful for patients with stage 4 and 5 CKD and mild proteinuria, in whom the presence of haematuria should not be neglected.

Our results suggest that moderate haematuria might be associated with all-cause mortality in patients with advanced-stage CKD. Hypertension and inflammation might be links. Hypertension, especially with a severe elevation in BP, could be associated with evidence of progressive target organ dysfunction, such as hypertensive nephropathy or myocardial infarction^32^. Haematuria is present in hypertensive nephropathy and is associated with fibrinoid necrosis or oedematous thickening of the intima in arterioles^28^,^33^. However, haematuria was not associated with mortality when creatinine was considered in patients with malignant hypertension^30^. Further evaluation of patients with CKD and malignant hypertension should be performed. Moreover, correlations were observed between haematuria and proteinuria, hypoalbuminemia, and an increased CRP, indicating an association between haematuria and inflammation status. Chronic inflammation in CKD is characterised by decreased renal function, arteriosclerosis, and glomerular and tubulointerstitial scarring^34^. Increased inflammation markers have been associated with kidney function, albuminuria, and CV events in patients with CKD^35^,^36^. Haematuria, through its association with inflammation, is likely associated with all-cause mortality. To the best of our knowledge, only one study included haematuria as a predictor of mortality in patients with biopsy-proven IgAN. The Japanese scoring system also includes haematuria as a predictor of mortality in patients with IgAN^37^. Therefore, additional studies are warranted to confirm the prognostic value of haematuria in predicting mortality.

In the study of Jeong *et al.*^38^, which enrolled 56,632 asymptomatic healthy adults in Korea, showed that female, older age, CKD and smoking were associated with microscopic hematuria while diabetes was associated with lower risk for hematuria. In the following study from this cohort^39^, the risk of significant underlying disease, such as renal stones, is higher in male and diabetes patients. In our study, we could not see these associations, probably because the effects of eGFR and proteinuria on hematuria are more important than other factors in CKD population.

## Limitations

This study has several limitations. First, although we collected urine samples at a steady status, and samples exhibiting >25 WBCs/hpf in urinalysis were excluded, asymptomatic urinary tract infections may have interfered with the haematuria assessment. Second, although participants exhibiting >50 RBCs/hpf in urinalysis were excluded, menstruation might have affected the results. However, we did not observe a correlation between haematuria and the female sex. Third, the origin of haematuria—glomerular or nonglomerular haematuria—was not ascertained in most of our patients, although amongst the 100 samples collected, most samples exhibited glomerular haematuria. Fourth, no consensus could be achieved on the definition of haematuria expressed as RBCs/hpf. We used the definition of ≥2–5 RBCs/hpf in 3 urinalyses, according to the definition reported in the largest cohort study^4^. Fifth, unlike in the quantification of albuminuria and proteinuria, which can be corrected using urinary creatinine, urine concentrations can interfere with the grading of haematuria severity. The severity of haematuria might not suggest a dose-dependent effect. Defining the exact amount of haematuria required to affect clinical outcomes is difficult. Sixth, extremely diluted or concentrated urine samples could cause haemolysis of the RBCs and underestimation of the severity of haematuria. However, the sensitivity test performed according to the dipstick occult blood method, which is used to measure haemoglobin or myoglobin, also revealed similar results. Seventh, although we arranged renal sonography to exclude urinary stones and malignancies, we did not arrange more sensitive studies for exclusion before study enrolment.

## Conclusion

Haematuria was significantly associated with increased risks of ESRD, rapid renal function progression, and all-cause mortality in patients with stage 3–5 nondiabetic CKD, particularly in those with mild proteinuria. Additional studies must be conducted to determine the role of haematuria in patients with nondiabetic CKD who have not undergone biopsy.

## Additional Information

**How to cite this article**: You-Hsien Lin, H. *et al.* Microscopic Haematuria and Clinical Outcomes in Patients With Stage 3-5 Nondiabetic Chronic Kidney Disease. *Sci. Rep.* **5**, 15242; doi: 10.1038/srep15242 (2015).

## Supplementary Material

Supplementary Information

## Figures and Tables

**Table 1:** Characteristics of patients with stage 3 to 5 non-diabetic CKD divided by haematuria

**Variable**	**All**	**Haematuria**			
		**No (0–2 RBCs/hpf)**	**Mild (2–5 RBCs/hpf )**	**Moderate (≧5–10 RBCs/hpf )**	***P*** **for trend**
No. of patients	1799	1019	508	272	*–*
**Demographics and medical history**					
Age (year)	62.6 ± 14.5	63.3 ± 13.8	62.7 ± 14.2	59.8 ± 16.8	<0.001
Sex (female)	757 (42.1%)	390 (38.3%)	241 (47.4%)	126 (46.3%)	0.001
Comorbidity					
Cardiovascular disease	326 (18.1%)	191 (18.7%)	83 (16.3%)	52 (19.1%)	0.763
Ischemic heart disease	193 (10.7%)	113 (11.1%)	46 (9.1%)	34 (12.5%)	0.906
Congestive heart disease	153 (8.5%)	88 (8.6%)	40 (7.9%)	25 (9.2%)	0.952
Cerebrovascular disease	206 (11.5%)	118 (11.6%)	56 (11.0%)	32 (11.8%)	0.963
Hypertension	1015 (56.4%)	595 (58.4%)	262 (51.6%)	158 (58.1%)	0.317
Smoker	181 (10.1%)	101 (9.9%)	49 (9.6%)	31 (11.4%)	0.584
Mean BP (mmHg)	99.8 ± 13.6	99.5 ± 13.3	99.9 ± 13.5	101.0 ± 14.9	0.112
BMI (Kg/m^2^)	24.4 ± 3.9	24.6 ± 3.9	24.1 ± 3.9	24.3 ± 4.3	0.265
**Renal function status**					
eGFR (ml/min/1.73 m^2^)	25.4 ± 15.7	27.7 ± 15.5	21.8 ± 14.7	23.6 ± 16.6	<0.001
UPCR (mg/g)	881 (333–1766)	661 (246–1349)	1197 (501–2139)	1324 (609–2655)	<0.001
**Laboratory data**					
Hemoglobin (g/dl)	11.1 ± 2.4	11.5 ± 2.5	10.6 ± 2.2	10.6 ± 2.5	<0.001
WBC (x1,000 cells/μl)	6.9 ± 2.2	6.8 ± 2.2	6.9 ± 2.2	7.2 ± 2.5	0.011
Platelet (x1,000 cells/μl)	211.0 ± 68.9	211.0 ± 67.1	213.3 ± 71.4	206.8 ± 70.4	0.371
Albumin (g/dl)	3.9 ± 0.5	4.0 ± 0.4	3.9 ± 0.5	3.7 ± 0.6	<0.001
Blood glucose (mg/dl)	104.6 ± 28.7	104.1 ± 26.6	106.5 ± 32.5	102.9 ± 28.4	0.535
ALT (mg/dl)	25.2 ± 23.9	25.5 ± 21.9	24.7 ± 26.6	25.4 ± 24.8	0.943
Total cholesterol (mg/dl)	190 (163–219)	189 (164–217)	189 (161–217)	195 (159–230)	0.337
Triglyceride (mg/dl)	119 (85–171)	116 (85–170)	120 (84–170)	119 (88–178)	0.280
CRP (mg/l)	1.1 (0.4–4.5)	0.8 (0.3–3.0)	1.5 (0.5–6.7)	1.6 (0.5–7.7)	<0.001
Sodium (mEq/l)	138.6 ± 3.4	138.8 ± 3.3	138.7 ± 3.5	138.2 ± 3.5	0.019
Potassium (mEq/l)	4.3 ± 0.6	4.3 ± 0.5	4.4 ± 0.6	4.3 ± 0.6	0.638
Phosphorus (mg/dl)	4.3 ± 1.3	4.2 ± 1.2	4.5 ± 1.3	4.6 ± 1.5	<0.001
Calcium (mg/dl)	9.1 ± 0.8	9.2 ± 0.7	9.0 ± 0.8	8.9 ± 0.8	<0.001
Bicarbonate (mEq/l)	21.6 ± 4.6	22.3 ± 4.3	20.6 ± 4.8	21.0 ± 4.6	<0.001
Uric acid (mg/dl)	7.8 ± 2.0	7.8 ± 1.8	7.9 ± 2.1	8.0 ± 2.1	0.143
**Clinical outcomes**					
Follow-up days	1157 (682–1768)	1217 (763–1809)	1215 (696–1760)	850 (486–1673)	<0.001
eGFR slope (ml/min/1.73 m^2^/year)	−1.7 (−4.4 to 0.1)	−1.2 (−3.4 to 0.5)	−2.2 (−5.1to –0.4)	−2.8 (−7.5 to –0.7)	<0.001
ESRD	507 (28.2%)	213 (20.9%)	203 (40.0%)	91 (33.5%)	0.005
Mortality	200 (11.1%)	91 (8.9%)	63 (12.4%)	46 (16.9%)	<0.001
CV events	196 (10.9%)	90 (8.8%)	73 (14.4%)	33 (12.1%)	0.010

CKD: chronic kidney disease, DM: diabetes mellitus, BP: blood pressure, BMI: body mass index, eGFR: estimated glomerular filtration rate, UPCR: urine protein to creatinine ratio, WBC: white blood cell, ALT: alanine aminotransferase, CRP: c-reactive protein, ESRD: end-stage renal disease, CV: cardiovascular.

Data are presented as mean  ±  standard error, median (interquartile range), or count (percentage).

**Table 2:** Multivariate logistic regression for haematuria.

**Variables**	**Odds ratio**	**95% CI**	***P*****-value**
Age (year)	0.994	0.987 to 1.001	0.113
Male vs. female	1.082	0.861 to 1.359	0.499
Cardiovascular disease	0.836	0.638 to 1.095	0.194
Mean BP (mmHg)	0.999	0.992 to 1.007	0.898
BMI (Kg/m2)	0.983	0.957 to 1.009	0.201
eGFR (ml/min/1.73 m^2^)	1.006	0.996 to 1.016	0.270
Log-transformed UPCR	2.242	1.747 to 2.876	<0.001
Hemoglobin (g/dl)	0.937	0.877 to 1.002	0.057
Albumin (g/dl)	0.654	0.514 to 0.832	0.001
Log-transformed cholesterol	1.277	0.469 to 3.480	0.632
Log-transformed CRP	1.361	1.205 to 1.536	<0.001
Phosphorus (mg/dl)	1.035	0.933 to 1.147	0.515
Uric acid (mg/dl)	1.009	0.956 to 1.065	0.739

DM: diabetes mellitus, BP: mean blood pressure, BMI: body mass index, eGFR: estimated glomerular filtration rate, UPCR: urine protein to creatinine ratio, CKD: chronic kidney disease, CRP: c-reactive protein, HbA1c: glycated hemoglobin.

*P* < 0.05 indicates a significantly associated with haematuria.

**Table 3:** Associations between haematuria and clinical outcomes.

	**Haematuria**			
	**No**	**Mild**	**Moderate**	***P*** **for trend**
***HR for ESRD***				
Unadjusted	1 (reference)	2.35 (1.94–2.85)**	2.30 (1.79–2.94)**	<0.001
Fully-adjusted	1 (reference)	1.28 (1.05–1.56)*	1.34 (1.03–1.74)*	0.024
***OR for rapid renal function progression***				
Unadjusted	1 (reference)	1.77 (1.31–2.39)**	2.48 (1.76–3.49)**	<0.001
Fully-adjusted	1 (reference)	1.45 (1.05–2.00)*	1.54 (1.06–2.25)*	0.023
***HR for all-cause mortality***				
Unadjusted	1 (reference)	1.59 (1.20–2.11)*	2.27 (1.64–3.14)**	<0.001
Fully-adjusted	1 (reference)	1.15 (0.86–1.54)	1.56 (1.11–2.20)*	0.039
***HR for CV events***				
Unadjusted	1 (reference)	1.63 (1.09–2.45)*	1.72 (1.26–2.34)**	0.001
Fully-adjusted	1 (reference)	1.06 (0.70–1.63)	1.33 (0.97–1.82)	0.204

HR: hazard ratio, OR: odds ratio, CV: cardiovascular. Rapid renal function progression is defined as eGFR slope < −5 mL/min/1.73 m^2^/year.

Model adjusts for age, gender, eGFR, log-transformed UPCR, hypertension, cardiovascular disease, mean BP, BMI, hemoglobin, albumin, log-transformed cholesterol, log-transformed CRP and phosphorus.

*(*P *< 0.05) or **(*P *< 0.01) indicates a significantly different from reference group; *P* for trend <0.05 indicates a significant trend for haematuria.

**Table 4:** Subgroup analysis of the association between haematuria and ESRD in the fully adjusted Cox proportional hazards model.

	**Haematuria**				
	**No**	**Mild**	**Moderate**	***P*** **for trend**	**P for Inter-action**
***All patients***	1 (reference)	1.28 (1.05–1.56)*	1.34 (1.03–1.74)*	0.024	
***UCPR subgroups***					0.048
***<500 mg/g***	1 (reference)	3.52 (1.22–10.14)*	4.41 (1.17–16.70)*	0.006	
***500–1500 mg/g***	1 (reference)	1.17 (0.86–1.76)	1.16 (0.87–1.72)	0.269	
***>1500 mg/g***	1 (reference)	1.16 (0.88–1.52)	1.32 (0.91–1.90)	0.307	
***CKD subgroups***					0.519
***Stage 3***	1 (reference)	1.67 (0.30–9.49)	1.48 (0.19–11.3)	0.830	
***Stage 4***	1 (reference)	1.55 (0.97–2.47)	1.47 (0.72–2.97)	0.165	
***Stage 5***	1 (reference)	1.26 (1.00–1.58)*	1.30 (0.97–1.75)	0.082	

Model adjusts for age, gender, eGFR, log-transformed UPCR, hypertension, cardiovascular disease, mean BP, BMI, hemoglobin, albumin, log-transformed cholesterol, log-transformed CRP and phosphorus.

*P < 0.05 indicates a significantly different from reference group; *P* for trend <0.05 indicates a significant trend for haematuria. *P* for interaction <0.05 indicates a significant modifying effect of the subgroup.
