# Supplementary material for: Microscopic Haematuria and Clinical Outcomes in Patients With Stage 3–5 Nondiabetic Chronic Kidney Disease
Source: Sci Rep. 2015 Oct 16;5:15242. doi: 10.1038/srep15242 (PMC4607953; doi:10.1038/srep15242)
Supplement: Supplementary Information [file srep15242-s1.pdf]

Microscopic Haematuria and Clinical Outcomes in Patients With Stage 3–5 Nondiabetic Chronic Kidney Disease

Hugo You-Hsien Lin[1,2,3], Chun-Yu Yen[1,6], Lee-Moay Lim[1], Daw-Yang Hwang[1], Jer-Chia Tsai[1,4], Shang-Jyh Hwang[1,4,5], Chi-Chih Hung[1], Hung-Chun Chen[1,4]

1. Division of Nephrology, Department of Internal Medicine, Kaohsiung Medical University Hospital, Kaohsiung Medical University
2. Department of Internal Medicine, Kaohsiung Municipal Ta-Tung Hospital, Kaohsiung Medical University
3. Graduate Institute of Medicine, College of Medicine, Kaohsiung Medical University, Kaohsiung, Taiwan
4. Faculty of Renal Care, College of Medicine, Kaohsiung Medical University, Kaohsiung, Taiwan
5. Institute of Population Sciences, National Health Research Institutes, Miaoli, Taiwan
6. Department of Pediatrics, Taipei Veterans General Hospital, Taipei, Taiwan, R.O.C.

Supplement Table 1. Cox regression for ESRD and mortality according to haematuria and urine occult blood

	Haematuria		
	**No**	**Mild+Moderate**	***P*- value**
HR for ESRD			
Unadjusted	1 (reference)	2.34 (1.96-2.79)**	<0.001
Fully-adjusted	1 (reference)	1.29 (1.07-1.56)*	0.007
HR for all-cause mortality			
Unadjusted	1 (reference)	1.80 (1.40-2.31)**	<0.001
Fully-adjusted	1 (reference)	1.28 (0.98-1.66)	0.067

	Urine occult blood		
	**No**	**+ ~ +++**	***P*- value**
HR for ESRD			
Unadjusted	1 (reference)	4.92 (3.78-6.39)**	<0.001
Fully-adjusted	1 (reference)	1.33 (1.01-1.75)*	0.041
HR for all-cause mortality			
Unadjusted	1 (reference)	1.44 (1.09-1.90)*	0.010
Fully-adjusted	1 (reference)	1.07 (0.79-1.44)	0.669

Model adjusts for age, gender, eGFR, log-transformed UPCR, hypertension, cardiovascular disease, mean BP, BMI, hemoglobin, albumin, log-transformed cholesterol, log-transformed CRP and phosphorus.

* ($P < 0.05$) or ** ($P < 0.01$) indicates a significantly different from reference group
